# Genetics research at the "Centenary of human population genetics" conference and SBB-2019

**DOI:** 10.1186/s12863-020-00906-7

**Published:** 2020-10-22

**Authors:** Tatiana V. Tatarinova, Ludmila E. Tabikhanova, Gilda Eslami, Haihua Bai, Yuriy L. Orlov

**Affiliations:** 1 Department of Biology, University of La Verne, La Verne, CA 91750 USA; 2 Department of Fundamental Biology and Biotechnology, Siberian Federal University, 660074 Krasnoyarsk, Russia; 3 N.I.Vavilov Institute of General Genetics RAS, 119991 Moscow, Russia; 4 Institute of Cytology and Genetics SB RAS, 630090 Novosibirsk, Russia; 5 Novosibirsk State University, 630090 Novosibirsk, Russia; 6 Research Center for Food Hygiene and Safety, School of Public Health, Shahid Sadoughi University of Medical Sciences, Yazd, 8916188638 Iran; 7 Department of Parasitology and Mycology, School of Medicine, Shahid Sadoughi University of Medical Sciences, Yazd, 8916188638 Iran; 8 Inner Mongolia University for the Nationalities, 028000, Tongliao, China; 9 The Digital Health Institute, I.M.Sechenov First Moscow State Medical University (Sechenov University), 119991 Moscow, Russia

Human population genetics has more than hundred year history. Modern next-gen sequencing technologies, genotyping and bioinformatics extended the research boundaries raising new genetics problems. This special issue of BMC Genetics presents the selected works studies discussed at the “Centenary of Human Population Genetics” conference in Moscow in May 2019, organized on the base of Moscow State University (http://www.centenary-popgene.com/). The Conference was focused on the discussion of the research on gene pools of the world’s nations, ancient DNA analysis, possibilities of judicial genetics, population-genetic database development, biobanks, and new genomics technologies. It was a unique event referred to as the 100th anniversary of the first human population study in 1919.

This journal special issue contains materials on human genetics and genomics presented at the conference in Moscow and SBB-2019 (Systems Biology and Bioinformatics - 2019) School in Novosibirsk in 2019 (http://conf.bionet.nsc.ru/sbb2019/en/). The SBB School series on bioinformatics is organized annually since 2008 by the Institute of Cytology and Genetics of the Siberian Branch of the Russian Academy of Sciences and Novosibirsk State University [1]. We have published selected conference materials as special issues at BMC Genetics and related BioMed Central journals [2–5].

The SBB Schools in Novosibirsk are satellite events for BGRS\SB (Bioinformatics of Genome Regulation and Structure \ Systems Biology) (https://bgrssb.icgbio.ru/2020) multiconference with genetics sessions [1, 6, 7]. The SBB-2019 School was held as a broad-scope independent meeting, genetics in topics with the human genomics and bioinformatics areas. The special issue on bioinformatics is accompanied by other BMC journal issues in the genomics, bioinformatics, and medical genetics areas at BMC Bioinformatics, BMC Genomics, BMC Medical Genomics, and BMC Medical Genetics, as well as in BMC Microbiology. We continued the BMC Genetics special issues in 2019 [2, 8, 9]. We believe such events and public discussion at the platforms of international publishers will bring the attention of the journal readers to actual genomics challenges.

We open up this Special Issue by the human population genetics study in Africa by the article by Sandra Walsh and colleagues [10] (this issue). African populations are genetically more diverse than any other human population, holding the highest amount of genetic variation, low linkage disequilibrium, and deep population structure. In the process of adaptation of humans to their environment, positive or adaptive selection has played a main role. Positive selection has been under-studied in African populations and less presented in such databases as 1000 Genome Project [11]. The authors used about hundred of available whole-genome sequences from five Ethiopian populations to investigate the modes and targets of positive selection. Walsh and colleagues found population-specific and shared signals of selection, with folate metabolism and the related ultraviolet response and skin pigmentation standing out as a shared pathway, possibly as a response to the high levels of ultraviolet irradiation, and in addition strong signals in genes such as IFNA, MRC1, immunoglobulins and T-cell receptors which contribute to defend against pathogens.

Maxat Zhabagin et al. [12] (this issue) present population genetics study in continental Asia. The authors analyzed the medieval Mongolian roots of paternal lineages from Kazakhstan. The majority of the Kazakhs from South Kazakhstan belong to the twelve clans of the Senior Zhuz. According to traditional genealogy, nine of these clans have a common ancestor and constitute the Uissun tribe [13]. The authors have genotyped 490 samples of South Kazakhs by set of Y-chromosomal SNPs. The Y-chromosomal variation in Kazakh clans indicates their common origin in medieval times, in agreement with the traditional genealogy [14]. Zhabagin and co-authors show that the Y-chromosomal lineages of South Kazakhstan were brought by the migration of the population related to the medieval Niru’un Mongols.

Work by Vladimir Babenko and colleagues [15] (this issue) analyze continental populations for variability in optical disk size morphology. *GFI1* (Growth Factor Independent Transcription Repressor 1) is a development gene which is likely to affect optic disk area by altering the expression of the associated genes via long-range interactions. Role of gene regulatory regions in the human genome were discussed in [16] as part of special post-conference Supplement issues at BioMed Central Distribution of haplotypes in the putative enhancer region has been assessed using the data on four continental super groups from the 1000 Genomes Project. The major haplotype appears to be involved in silencing *GFI1* repressor gene expression, which might be the cause of increased optic disk area characteristic of the East Asian populations.

Ramjet Das and Priyanka Upadhyai [17] analyzed the *Kumhar* and *Kurcha* populations from the India. The authors investigated the genetic origin and population history of the *Kumhars*, a group of people who inhabit large parts of northern India using the geographic population structure method developed earlier [18]. Das and Upadhyai compared 27 previously published *Kumhar* SNP genotype data sampled from Uttar Pradesh in north India to various modern day and ancient populations. The analysis show high genomic proximity to the *Kurchas*, a small and relatively little-known population found far away in Kerala, south India. The findings illuminate the genomic history of two Indian populations, allowing a glimpse into one or few of numerous of human migrations that likely occurred across the Indian subcontinent [19].

Rosa Tiis and co-authors [20] (this issue) studied polymorphic variants of the NAT2 (N-acetyltransferase 2) gene in native populations of Siberia. NAT2 plays a crucial role in the metabolism of a wide range of xenobiotics, including many drugs, carcinogens, and other chemicals in the human environment [21]. This work presents for the first time data on the frequency of two variants of NAT2 gene, which significantly affect the rate of xenobiotics acetylation, among the representatives of indigenous populations of Forest and Tundra Nenets in Northern Siberia. Genetic predispositions to diabetes and related diseases among native Mongolian populations were studied by the authors’ group earlier in [22, 23].

The work by Mikhail Ponomarenko and colleagues [24] (this issue) covers fundamental evolution problems of natural selection by male reproductive potential. The concept of reproductive potential denotes the most vital indicator of chances to produce and sustain a healthy descendant. The authors continued study on single-nucleotide polymorphisms (SNP) in TATA-binding protein binding sites in human gene promoters [25, 26]. The authors found in silico new candidate SNP markers of male reproductive potential.

The other works consider genetics application in model organisms. These articles discuss genetics application in Drosophila, highlighted, in turn, at SBB’2019 School in Novosibirsk.

Anna Ogienko et al. [27] (this issue) analyzed Drosophila lines for Gal4 gene. The authors provide a mini-atlas of the spatial activity of Gal4 drivers that are widely used for the expression of UAS genes in the Drosophila [28, 29].

Mikhail Shaposhnikov and co-authors [30] (this issue) consider problems of aging and affecting life span on fruit flies. Beta-amyloid peptide (Aβ) is the key protein in the pathogenesis of Alzheimer’s disease, the most common age-related neurodegenerative disorder in humans. The authors used the *Drosophila* model [31] to study mechanisms underlying a dual role for Aβ peptides.

The work by Natalia Blazhko et al. [32] concludes this issue by analysis of virulence properties for *bovine leukemia virus*. This study describes the biodiversity and properties of this virus in Western Siberia. The paper explores the effect of different genotypes of the *env* gene of the cattle leukemia virus on hematological parameters of infected animals. The authors note that monitoring the origin of new virus mutations is of great importance for veterinary and animal husbandry, as every new strain may have unique features of interaction with the host organism. The problems of hazards control in food safety related to infectious diseases became important due to the SARS-CoV-2 pandemic [33]. Genetics studies on model organisms have new value in relation to the infectious disease resistance, adaptations of human populations to environment, and natural polymorphism.

We aim to support international exchanges and education in the form of international conferences and schools for young scientists on bioinformatics, genetics and systems biology [1] (https://peerj.com/collections/72-bgrs-sb-2020/). We invite our readers worldwide to attend the systems biology meetings in Russia - Digital Medicine Forum and MGNGS-2020 (Medical Genetics - Next-Generation Sequencing) event postponed to 2021 (http://ngs.med-gen.ru/mgngs20/).
